# Virtual Reality As a Training Tool to Treat Physical Inactivity in Children

**DOI:** 10.3389/fpubh.2017.00349

**Published:** 2017-12-20

**Authors:** Adam W. Kiefer, David Pincus, Michael J. Richardson, Gregory D. Myer

**Affiliations:** ^1^Division of Sports Medicine, Cincinnati Children’s Hospital Medical Center, Cincinnati, OH, United States; ^2^Department of Pediatrics, College of Medicine, University of Cincinnati, Cincinnati, OH, United States; ^3^Center for Cognition, Action and Perception, University of Cincinnati, Cincinnati, OH, United States; ^4^Department of Psychology, Chapman University, Orange, CA, United States; ^5^Department of Psychology, Macquarie University, Sydney, NSW, Australia; ^6^The Micheli Center for Sports Injury Prevention, Waltham, MA, United States

**Keywords:** virtual reality, neuromuscular training, perceptual-motor learning, obesity prevention, physical inactivity

## Abstract

Lack of adequate physical activity in children is an epidemic that can result in obesity and other poor health outcomes across the lifespan. Physical activity interventions focused on motor skill competence continue to be developed, but some interventions, such as neuromuscular training (NMT), may be limited in how early they can be implemented due to dependence on the child’s level of cognitive and perceptual-motor development. Early implementation of motor-rich activities that support motor skill development in children is critical for the development of healthy levels of physical activity that carry through into adulthood. Virtual reality (VR) training may be beneficial in this regard. VR training, when grounded in an information-based theory of perceptual-motor behavior that modifies the visual information in the virtual world, can promote early development of motor skills in youth akin to more natural, real-world development as opposed to strictly formalized training. This approach can be tailored to the individual child and training scenarios can increase in complexity as the child develops. Ultimately, training in VR may help serve as a precursor to “real-world” NMT, and once the child reaches the appropriate training age can also augment more complex NMT regimens performed outside of the virtual environment.

The high prevalence of overweight and obese children continues to be a major public health problem (1). Myriad factors underlie this etiology, with physical inactivity identified as a primary cause (2), and one potential mechanism is that many children are not developing the motor skill repertoire and movement confidence at a rate necessary to initiate and maintain a healthy level of physical activity (3, 4). Specifically, both body mass index and physical activity have shown to be predictive of individuals’ Functional Movement Screen scores (5). Further, childhood motor skill proficiency has demonstrated to be predictive of adolescent physical activity (6, 7). While there is limited evidence that there exists a direct link between motor skill proficiency and motor skill confidence, it is likely that motor skill competency is accompanied by motor skill confidence, with the latter a possible mediator in the relation between motor skill competency and obesity. Neuromuscular training (NMT) is an established conceptual program designed to fill this void and improve physical activity in children. NMT is designed to enhance health-related and skill-related components of physical fitness that facilitate safe, effective, and developmentally appropriate fitness training for school-age children (8–10). Specifically, NMT is a focused set of motor control and strength building activities that promote the development of a child’s neurocognitive and perceptual-motor capabilities and, ultimately, ameliorates fundamental motor skill deficits to promote movement confidence. Movement confidence and competence are necessary to promote increases in physical activity and build interest in behaviors (11) that, combined, meet the 60 min or more of moderate to vigorous physical activity, which make up current public health recommendations.

Importantly, NMT-based skill development and retention requires that the child’s level of cognitive and perceptual-motor development is at a level appropriate for individualized programming—typically around 6 or 7 years of age (10). Appropriate programming for children is also conditional on the child’s *training age*—an age that represents the child’s prior experiences of context-specific training and driven, in part, by the child’s natural development. The initiation of training at an earlier age relates to an increased likelihood of avoiding physical inactivity later in life (12, 13). Thus, an obvious solution is to start the training age “clock” earlier using NMT to advance the time course of motor skill proficiency. This is not easy, however, as the introduction of the child to training is dependent on whether the child is physically or mentally prepared for formalized and specialized training at an earlier age. Therefore, perceptual-motor skill development may be a limiting factor for earlier initiation of NMT. Thus, new interventions are needed to circumvent this training barrier.

## *In Virtuo* Training Scenarios

Virtual reality (VR), or *in virtuo*,^1^ training is a viable option to manage limitations inherent to implementation of NMT in children in the early phases of perceptual-motor skill development. The advantage of VR-based training over more standard real-world training interventions (15–19) is that VR can leverage known principles that underlie an information-based behavioral account of motor development (20, 21), and this does not necessitate more advanced phases of perceptual-motor skill developmental that are prerequisites of NMT and other formalized activities. An information-based account presupposes that proficient motor performance arises *via* unique, learned relations between the information available to the child *via* their environment and the biomechanical properties of the motor system as children assemble their bodies in specific ways to complete a given task (22–24). Consider that from the time a child is born, the cognitive-perceptual-motor systems are honed to capitalize on action possibilities in the environment. Over time, children learn to exploit information about their bodies (e.g., visual, proprioceptive, and other sensory information about limb and body size and position; muscle strength and biomechanics) as well as information about the world around them (e.g., the natural physics of the world and of objects with which they interact) as they move through and act on their environment. The success and failure of these endeavors provide children feedback as they learn the extent of their action capabilities and ultimately lead to the development of goal-directed behaviors. Through the course of normal development, the child executes tasks of greater complexity as he/she learns to combine individual movements into strings of movement patterns to achieve task goals and, eventually, strings of task goals. Thus, every behavior is a set of perception–action feedback loops that shape the child’s time course of natural development and serves as the dynamic substratum for how children interact with the world around them.

Conversely, a child with motor-skill deficits and/or low movement confidence may struggle to take full advantage of these types of perception–action relations and, as a result, may perform movements more slowly, or default to more basic, early stage movement patterns compared to their typically developing peers (e.g., consider a stage two throw). Such performance deficits may be exacerbated as these children observe their peers performing more and more complex tasks with greater success, while the children with lower motor skill proficiency toil in movement patterns that are suboptimal for adequate performance gains. This can further diminish movement confidence and can lead to a cascade of movement deficits that underlie reduced physical activity, and that will lead the child down the path toward obesity later in life. VR training can integrate the necessary information-based interventions into simulated play in such a way that the child can learn to exploit these perception–action relations at their own pace to optimize motor skill learning. The potential to jump-start the movement-deficient child with fully immersive scenarios can build confidence in movement strategies in a safe and “fun” environment.

## Fully Immersive Virtual Environments (FIVEs)

Virtual reality-based training that targets perception–action relations mandates certain requirements for effective implementation, with *presence* the most important for successful implementation. *Presence* is achieved when the VR experience compels a feeling of being present in an environment other than the real, physical environment in which the individual is actually performing (25). Presence is increased through the development of specific immersion criteria within a given scenario. Here, we define a FIVE as one made up of five immersion criteria (see Table 1): (1) physical, (2) spatial, (3) strategic, (4) social, and (5) narrative. Similar to Weiss et al. we define *general immersion* as the extent to which the scenario refocuses the child’s sensations and perception from the real world to the virtual one (26), with all five criteria integral to the achievement of that goal.

**Table 1:** Examples of essential criteria for a fully immersive virtual environment training scenario.

	Physical immersion	Spatial immersion	Strategic immersion	Social immersion	Narrative immersion
Description	Ambulatory movements in the physical world	Perceptually convincing physics	Facilitates physical and cognitive responses	Interaction with other non-player characters	Promotes investment in the virtual reality scenario

Example	Real-scale movement with no cables or wires	Optic flow or haptic feedback	Artificial intelligence and strategy	Interaction with avatars and virtual humans	Story behind the scenario

*Physical immersion* is accomplished through an ambulatory, three-dimensional (3D) environment that allows the child to navigate the virtual world by walking or running through the virtual (and real) space (27)—with real scale movement especially important for the physically inactive child. *Spatial immersion* is characterized by a virtual world that is perceptually convincing to the child: that is, objects are of similar scale and move as they would in the physical world (i.e., natural physics) and the child perceives her movement to match that of the real world based on believable optical information, or *optic flow*—the pattern of motion available from the ground and background surfaces that specifies information about movement speed and direction (28, 29). *Strategic immersion* requires a combination of immediate, physical responses with more cerebral, cognitive decisions adopted to complete scenario related tasks. This can be enhanced through adaptive artificial intelligence (AI) and well-designed scenarios will require both types of stratagem for successful performance. The fourth component is *social immersion* and relates to the child’s interaction with other non-player characters (NPCs) that are present in the virtual world. Additional children or an instructor may drive this type of character—commonly referred to as *avatars—*and these humans may or may not share the same real-world physical space as the child. More likely, these characters are driven by AI models of behavior, and thus termed *virtual humans*. Importantly, if it is difficult for the child to tell the difference between a human-driven avatar and a virtual human, the social immersion is high. Finally, *narrative immersion* involves the child’s investment in the scenario or how much he/she cares about the additional NPCs and overall task goals. This is the story for the scenario and serves to motivate the child to perform energetically and in a way that can translate to the real world. Clinical-based VR interventions can be successful in the absence of some of these criteria (26). However, together, these components provide a foundation for the development of FIVE scenarios that can ground the child in a learning-rich *in virtuo* environment and require physical interaction to promote the transfer of learned skills to real world activities (see Table 2).

**Table 2:** A comparison of classic neuromuscular training (NMT) and fully immersive virtual environment (FIVE) integrated NMT.

	NMT	FIVE integrated NMT
Initiation	6 years of age	4 years of age
Instruction	Instructor dominant	Minimal Instruction
Development	Instruction based	Naturally induced
Motor skill learning	Instructor paced	Self paced
Feedback	In-between	Real time
	Movements	

A scenario that meets these five criteria can become extremely complex from both a hardware integration and virtual environment design standpoint. The former necessitates a head-mounted display (HMD) with a wide (≥100°) field of view for visualizing the 3D virtual environment. The HMD also needs to be wireless (i.e., untethered) with appropriate child-sized head straps, mounts, and padding for visual stability during active movements. A wireless degree of freedom positional tracking system should be used in conjunction with the HMD to provide a 1:1 mapping between the child’s position in the real and virtual environments. Failure to do so will result in a disruption of the child’s sense of realness in the virtual environment, and this can lead to feelings of physical disorientation and nausea (30). Accordingly, such a system requires a large physical space for the child to move—a 5 m × 5 m space is a minimum recommendation—to promote full-form movements and a physically active experience. Additional hardware technologies that provide tactile information (e.g., haptic devices) can provide additional feedback to the child as he or she interacts with the virtual world.

The remaining immersion criteria (strategic, social, and narrative) are more complex from a design standpoint. The development of 3D models that make up the environment can be tedious and expensive; however, well-designed environments, ground textures, and non-player character meshes can greatly enhance the strategic, social, and narrative immersion for the child. Similarly, NPC context specific movements require smooth animations, with true virtual humans requiring precursory motion capture animations of real, age-specific participants, and avatars requiring high fidelity motion capture with minimal lag times for real-time movement display. These animated movements also need to be accompanied by behaviors that are believable within the broader training context.

Complex AI can drive larger scale behavioral networks of artificial agents that move through the world and interact with it, each other, and the child in a believable way. These behavioral networks enhance the strategic immersion of the scenario by facilitating highly specific NPC responses to the user. Non-player character voice recognition also enhances the social immersion and provides the child a more natural means for communication with such characters. These types of non-player characters make up a key component of the virtual environment and establish a believable narrative that engages the child. Together, these five components (physical, spatial, strategic, social, and narrative) contribute to the overall immersion that the child experiences and facilitate the achievement of motor skill development goals.

## Initiate an Earlier Training Clock with Fives

In consideration of an information-based behavioral account, the initiation of training at an early age requires an intervention that can target the developing child at the level of information (i.e., feedback) to catalyze motor skill proficiency. VR is uniquely equipped for this and, specifically, can be used to artificially modify visual feedback the child receives as he/she moves through the virtual and real-world space. Thus, the VR scenario covertly modulates the child’s producible motor patterns in a similar way to how a child learns perceptual-motor relations while moving in the real world (i.e., implicit rather than explicit training). This training strategy is not without precedence. The manipulation of visual information through artificial visual displays is consistently effective in changing adult behavior (22, 31–40). For example, artificially manipulating the information that specifies the rotational kinematics of a hand-held object (i.e., decoupling the inertial gain) to optically specify a faster movement of the object on a virtual display while the wielder’s actual movements are hidden from view results in the wielder perceiving a lighter object, and vice versa (39, 40). Analogously, altering the rate of optic flow elicits changes in the walking speed of healthy adults by dissociating the biomechanics of their gait from their perception of walking speed (22). The pattern of optic flow can thus be shifted to the left or right and participants exhibit compensatory postural and steering adjustments that can cause them to walk to the left or right of an intended target (35, 36). This is consistent with the highly successful external focus of attention approach that is well supported in the motor learning literature (41–43).

Such visually induced changes to perception of heaviness and walking speed provide the initial theoretical foundation for the general *in virtuo* training, we propose to optimize training scenarios for children. Through the utilization of FIVE scenarios, a training regimen can be developed that considers the motor skill deficiencies of the individual child and affects change to their motor patterns gradually over the time course of the prescribed training. The process would likely begin with an assessment of the child’s overall movement capabilities and propensity for physical inactivity. Once completed, several FIVE scenarios could be developed to improve on the child’s existing deficits. These scenarios might involve fundamental movements (e.g., squatting, running, cutting, jumping, or landing) performed in a realistic environment within the context of structured play such as a virtual game of hopscotch, avoiding NPCs in a game of tag, or even chasing the random, 3D movement of a fluttering butterfly. During such tasks, the visual information that pertains to the child’s movement (e.g., optic flow, the movements of virtual limbs, the eye height of the child) in the virtual environment is altered in such a way that the child naturally and subconsciously slows their movements while simultaneously honing their perceptual-motor relations. These types of scenarios would allow the child to refine movement patterns at their own pace, during play, and with only minimal interaction with an instructor. This also allows for scenario complexity to increase during training while building the child’s confidence and increasing her motivation instead of overwhelming the child with difficult movements. Consider that a child learns much of her movement skills in a rich, complex world of information, objects, and people throughout normal development. FIVE scenarios can be designed in a similar way, with added situational complexity used to increase cognitive demands without burdening the child with extremely difficult tasks that require movements beyond the child’s capabilities. As training in these scenarios progresses, the visual information can gradually change until it matches the optic flow and the speed of limb movements in the real world. The progression of this may require several FIVE scenarios across many training sessions, as a precursor to formalized NMT. In this light, a child with a reduced training age might be able to “catch up” to his or her peers through FIVE-enhanced motor skill development, as the goal for FIVE scenarios is to support active reintegration of the child into real-world scenarios with their peers.

## Integrating Fives with NMT

Neuromuscular training and FIVE scenarios do not have to be mutually exclusive. As training progresses in FIVE scenarios, NMT-specific exercises can be introduced to the child, initially at a very rudimentary level, to build a foundation of task-specific training. At this point, feedback can be given to the child *in virtuo* to begin preliminary training of technique. For example, the visual information could be adapted in real time so that the next time the child lands from a jump the visual information about his/her height subtly changes to encourage a deeper knee bend during landing. As the child develops proficiency in certain skills, a weaning off period can begin. Here, the child could be gradually introduced to real-world NMT, and the FIVE scenarios will slowly transition from a primary training tool to a NMT supplement. This begins what has been previously termed a *transfer phase* (26)—the transference of learned skills from *in virtuo* to the real world. Throughout this process, a specialized educator or instructor becomes more and more directly involved during actual training, as technique begins to become a focus and training starts to resemble classic NMT approaches (10, 44).

As the child matures and NMT becomes a more prominent part of their overall training regimen, FIVE scenarios can evolve into active play to assess transfer. At this stage, the educator or instructor can become an NPC in the environment as well and provide instructions, in real time, to the child. Alternatively, a pre-recorded instructor can be utilized to provide specific reminders of motor skill technique prior to the beginning of a scenario. Other human or virtual-human training partners can also be introduced to initiate peer-to-peer, or dyadic, scenarios. These types of *in virtuo* interpersonal training scenarios provide several advantages over dyadic training in the real world. There is the potential for the child to have increased motivation to be physically active when interacting with additional characters. There is also the opportunity to utilize additional developmental learning strategies such as mimicry, or pattern matching, in which the child can emulate another character in the virtual world. It can also promote the development of pro-social behavior that may be transferable to real-world interactions between children and their peers. This training, combined with the increased confidence that is gained with fundamental motor skill development, may provide further motivation to promote a consistently active lifestyle for the child into the future. As technologies are developed that are more portable and cost-effective, *in virtuo* interventions will become more widely available and should become prominent tools for the fight against physical inactivity and, ultimately, obesity.

## Advantages and Considerations for *In Virtuo* Training

As described, there are many motor-learning advantages to training in VR instead of, or in supplement to, real-world training. In addition to those benefits, VR also may provide a lower-cost solution compared to instructor-led programs. Such programs could leverage VR technology to allow for the training of more children for a given session, with AI-based feedback driving within-trial or within-exercise changes that challenge the child appropriately. Performance data would be available in real-time for the instructor, and the instructor could provide additional feedback as needed. A similar strategy could be used in a telehealth setting for monitoring in-home training while the children are in between on-site training sessions. Importantly, VR training can provide a more fun and engaging environment, compared to real-world NMT. Based on adaptive feedback and, in following, the rules for FIVE scenario implementation, the training can be gamified to promote motivation, engagement, and enjoyment beyond that of traditional physical activity (45). While we have suggested similar interventions for real-world NMT (46), technology-integrated gamification would allow for an additional level of immersion and a fine-grained tuning of parameters that would allow for a more complete integration of fundamental training concepts with aspects of play.

There are, of course, also limiting factors that must be taken into consideration prior to implementing VR in young populations. First, there is currently no scientific basis for the safety and efficacy of VR in young children. Not unexpectedly, this has led to a lack of consensus among experts as to an appropriate age for existing VR HMD use: current hardware manufacturers differ on their safety language, with Oculus Rift (Oculus VR, LLC) setting an age limit of 13+ years, Sony Playstation VR (Sony Interactive Entertainment, LLC) setting an age limit of 12 years, and while the HTC Vive does not set an age limit, there is language in the user guide that states that the HTC Vive (HTC Corp.) was not designed to be used by children. However, several studies have demonstrated the utility of HMD-based VR systems for use in children as young as 5 years of age (47–51), and while safety was not a central focus of these studies, there were minimal to no side effects from HMD use in the young populations. Moreover, while there is currently no data on risks or contraindications from time spent in VR, as with TV/mobile screens, it is likely important to limit VR screen use to targeted training sessions of reasonably limited duration. For example, the training sessions advocated for in the current paper are designed to last for no more than 1.5 h (52), with VR training sessions as described here lasting no more than 30–45 min. While dosage limits and effects will need to be studied to fully understand training benefits and transfer, traditionally, NMT is only performed 3 days/week and, thus, screen time in VR would be far less than what the child would receive during normal daily activities (i.e., 2.5 h/day) (53). Another consideration is the design and implementation of headsets that fit a variety of head sizes. Just as a FIVE scenario requires a headset of specific design standards, adjustable straps and fittings are essential for comfort and safety in younger populations. In addition, HMD weight becomes more of a limiting factor for smaller users. Importantly, these are solvable engineering issues and will likely become more prevalent on future hardware as more and more younger users adopt VR technologies.

## Summary

Fully immersive virtual environment scenarios have the potential as important tools in the prevention and treatment of physical inactivity; however, much more work is needed to utilize them efficiently and effectively. Many outstanding issues still need to be addressed to understand the efficacy of FIVE scenarios for use with children under the age of 7 and how to appropriately design and implement visual environments to be most effective for training. The information-based framework for understanding perceptual-motor behavior (20, 21, 23, 29, 54) provides a strong foundation for training motor skills in children. It is also, perhaps, the most critical component to *in virtuo* training as it takes advantage of the relation between motor control and perceptual information. However, many open questions remain and addressing them requires a strong methodological approach grounded in theory to tease apart these relations. This will help researchers and educators better understand what types of visual information are most important for affecting change in the movement of children; an approach that is equally true for training in the real world. Ultimately, FIVE scenarios as a precursor to, and integrated with, NMT can be additive in the promotion of a healthy, active lifestyle in children and for the treatment of physical inactivity *via* increased confidence and enjoyment of physical activity during critical phases of motor skill development.

## Author Contributions

AK led the development of the manuscript and was primarily responsible for idea development and all writing. GM contributed to the exercise-deficit disorder and neuromuscular training components of the manuscript. DP assisted with idea generation and the interpersonal and cognitive development ideas and writing. MR assisted with the technology development sections and the information-based approach as integrated into the current manuscript.

## Conflict of Interest Statement

The authors declare that the research was conducted in the absence of any commercial or financial relationships that could be construed as a potential conflict of interest. The reviewer JN and handling editor declared their shared affiliation.

## Footnotes

^1^*In virtuo* (in virtual) is analogous to *in vivo* (in the living) and *in vitro* (in glass). It was first introduced by Jacques Tisseau (14). It represents human participation in the conditions of virtual reality and virtual environments.
